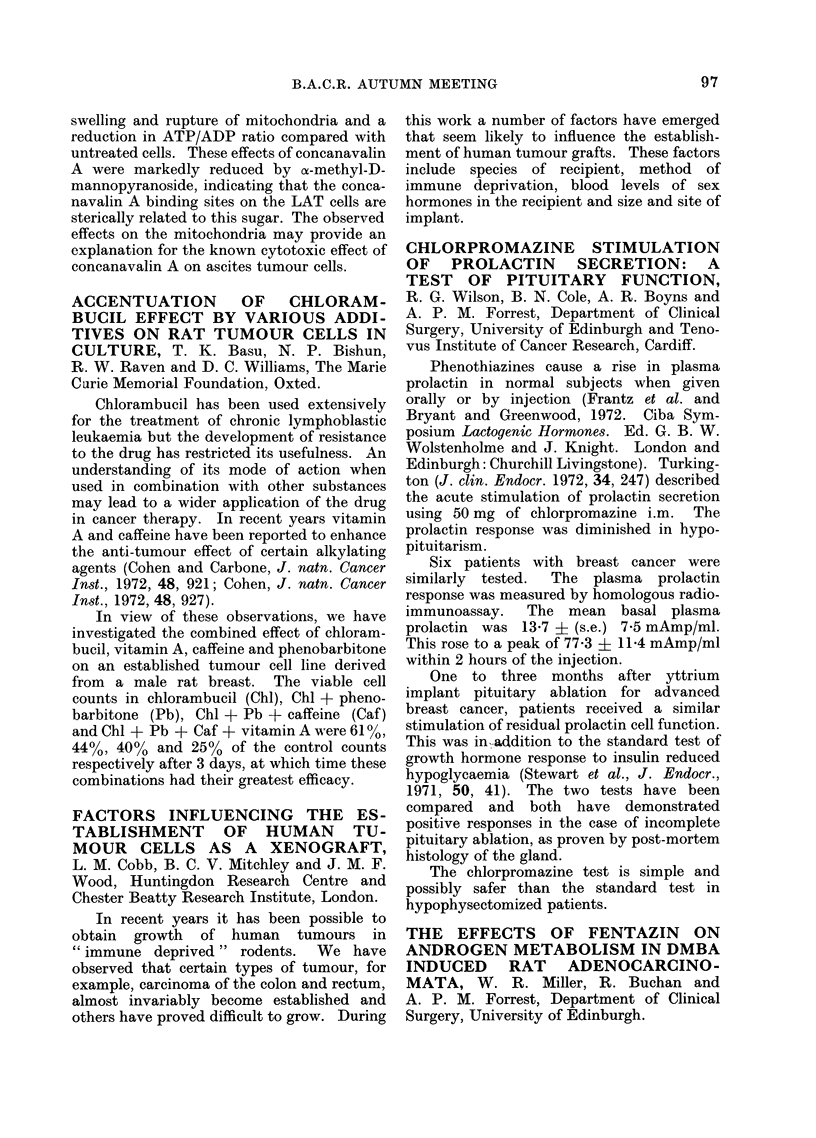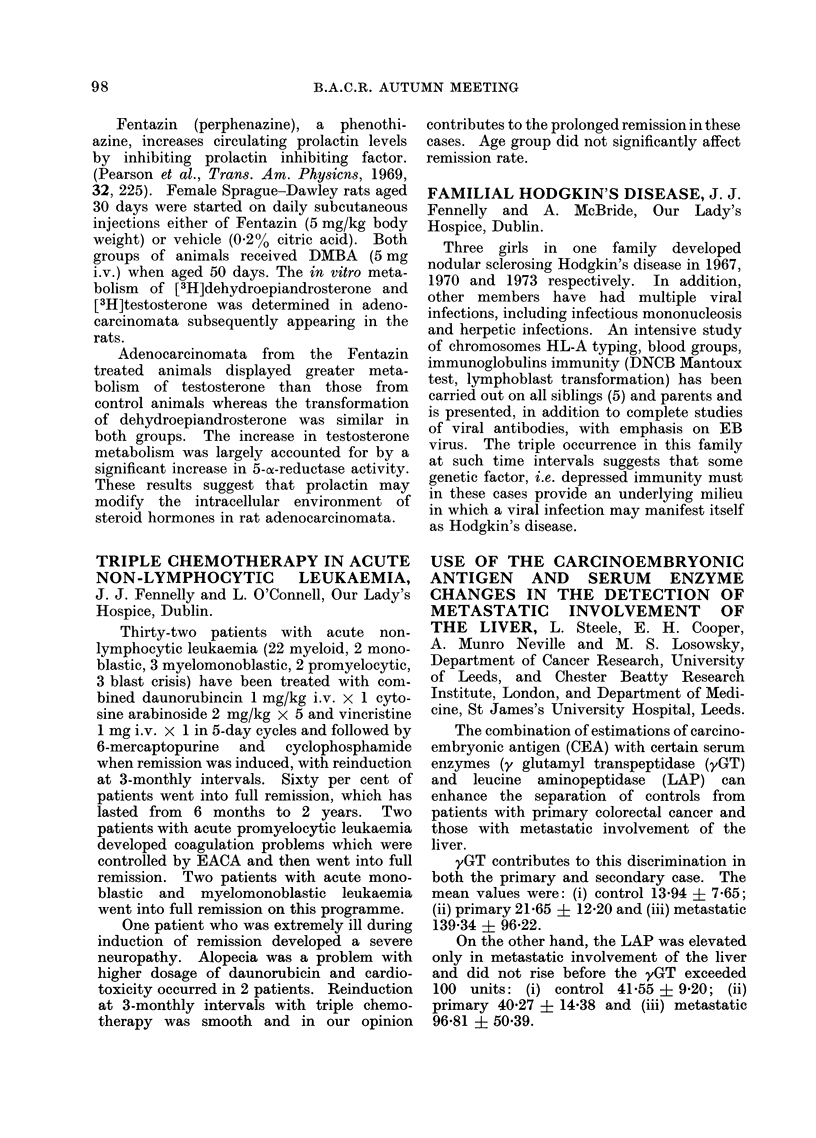# Proceedings: Chlorpromazine stimulation of prolactin secretion: a test of pituitary function.

**DOI:** 10.1038/bjc.1974.33

**Published:** 1974-01

**Authors:** R. G. Wilson, B. N. Cole, A. R. Boyns, A. P. Forrest


**THE EFFECTS OF FENTAZIN ON ANDROGEN METABOLISM IN DMBA INDUCED RAT ADENOCARCINOMATA,** W. R. Miller, R. Buchan and A. P. M. Forrest, Department of Clinical Surgery, University of Edinburgh.

Fentazin (perphenazine), a phenothiazine, increases circulating prolactin levels by inhibiting prolactin inhibiting factor. (Pearson *et al.*, *Trans. Am. Physicns*, 1969, **32**, 225). Female Sprague–Dawley rats aged 30 days were started on daily subcutaneous injections either of Fentazin (5 mg/kg body weight) or vehicle (0·2% citric acid). Both groups of animals received DMBA (5 mg i.v.) when aged 50 days. The *in vitro* metabolism of [$^3$H]dehydroepiandrosterone and [$^3$H]testosterone was determined in adenocarcinomata subsequently appearing in the rats.

Adenocarcinomata from the Fentazin treated animals displayed greater metabolism of testosterone than those from control animals whereas the transformation of dehydroepiandrosterone was similar in both groups. The increase in testosterone metabolism was largely accounted for by a significant increase in 5-$\alpha$-reductase activity. These results suggest that prolactin may modify the intracellular environment of steroid hormones in rat adenocarcinomata.